# Effectiveness of GLP‐1RA according to different type 2 diabetes phenotypes: A retrospective study

**DOI:** 10.1111/dom.70005

**Published:** 2025-08-11

**Authors:** Irene Caruso, Fiorella Giordano, Immacolata Ilaria Matichecchia, Ludovico Di Gioia, Sergio Di Molfetta, Angelo Cignarelli, Gian Pio Sorice, Annalisa Natalicchio, Luigi Laviola, Francesco Giorgino

**Affiliations:** ^1^ Department of Precision and Regenerative Medicine and Ionian Area, Section of Internal Medicine, Endocrinology, Andrology and Metabolic Diseases University of Bari Aldo Moro Bari Italy

**Keywords:** GLP‐1 receptor agonists, phenotypes, type 2 diabetes

## Abstract

**Aims:**

Type 2 diabetes (T2D) is a heterogeneous disease, with varying pathogenetic mechanisms influencing treatment response. Glucagon‐like peptide‐1 receptor agonists (GLP‐1RA) offer clear metabolic benefits in T2D, but interindividual variability in response is observed. This retrospective study investigated whether GLP‐1RA effectiveness varies by T2D phenotypes (severe insulin‐deficient diabetes [SIDD], severe insulin‐resistant diabetes [SIRD], mild obesity‐related diabetes [MOD], mild age‐related diabetes [MARD]) and assessed predictors of HbA1c reduction.

**Materials and Methods:**

Individuals attending the Endocrinology Unit at the University Hospital Policlinico Consorziale in Bari, Italy, between 01 January 2014, and 31 August 2024, were evaluated for participation. T2D phenotypes were assigned using the algorithm by Bello‐Chavolla. The primary outcome was the difference in HbA1c change from baseline among phenotypes, and predictors were identified using SHAP and multivariable regression. Time to treatment failure (HbA1c >7.0%) was estimated with Kaplan–Meier analysis.

**Results:**

Among 181 patients, SIDD individuals yielded a significantly greater HbA1c reduction (−2.6%[1.9]) versus other phenotypes (*p* < 0.0001). Baseline HbA1c was the strongest predictor of HbA1c reduction. SIDD was linked to faster treatment failure (HR 3.69, 95% CI 1.37–9.94;*p* < 0.01), but this effect disappeared after adjustment for baseline HbA1c.

**Conclusion:**

This study adds to the emerging evidence against the hard clustering approach to T2D phenotyping in terms of response to treatment and time to treatment failure, particularly with GLP‐1RA.

## 1 INTRODUCTION

Current recommendations on the management of patients with type 2 diabetes (T2D) take into account HbA1c and body weight (BW) targets, cardiovascular (CV) risk factors, comorbidities and safety profile as well as cost and availability of diabetes drugs.^1^ Apart from aiming at BW loss in patients with obesity, treatment choices are not informed by the specific pathophysiological processes causing diabetes in any given patient.^2^ Glucagon‐Like Peptide‐1 Receptor Agonists (GLP‐1RA) are among the most efficacious drugs for diabetes management, displaying significant glucose and BW lowering effects and cardio‐renal protection.^3^ However, randomized clinical trials (RCT) showed great heterogeneity in response to treatment, with up to approximately 30% of patients failing to achieve generally accepted targets.^2^, ^4^ Indeed, individuals with T2D differ in clinical features such as prior glycaemic control, BW and fat distribution, age at diabetes diagnosis and lipid profile, which may reflect differences in the underlying pathophysiology of the disease and affect prognosis and response to treatment.^5^

Contrariwise, precision medicine in T2D aims to assign specific glucose‐lowering therapies to the individuals most likely to benefit from them.^6^ Various approaches have been proposed to address T2D heterogeneity, such as the hard clustering approach by Ahlqvist et al., where individuals are assigned to a specific phenotype (mild age‐related diabetes [MARD], mild obesity‐related diabetes [MOD], severe insulin‐deficient diabetes [SIDD], severe insulin‐resistant diabetes [SIRD], and severe autoimmune diabetes [SAID]) based on age, HbA1c level, Body Mass Index (BMI), presence of GAD‐65 antibodies, HOMA2‐B and HOMA2‐IR (C‐peptide‐based Homeostasis Model Assessment for insulin secretion and resistance) at diagnosis.^7^ This approach is one of the most replicated; however, there is limited data on the value of this classification on response to treatment.^8^ The analysis of the datasets of the ADOPT and RECORD trials showed a preferential benefit of sulfonylureas in MARD and of thiazolidinediones in SIRD patients.^9^ A reanalysis of the ORIGIN trial demonstrated that SIDD individuals exhibited the greatest HbA1c reduction following treatment with insulin glargine.^10^ Dwibedi et al. recently investigated the effects of the GLP‐1RA semaglutide and sodium‐glucose cotransporter‐2 inhibitor (SGLT‐2i) dapagliflozin in SIDD and SIRD individuals in a randomized controlled trial,^11^ while there is no evidence to date on GLP‐1RA efficacy across the four T2D phenotypes.

In this study, we aimed to assess whether assignment to the specific diabetes phenotypes of non‐autoimmune diabetes previously identified by Ahqlvist et al. might affect response to GLP‐1RA and time to treatment failure.

## 2 METHODS

This was a single‐centre, observational, retrospective study conducted at the Division of Endocrinology of the University Hospital Consorziale Policlinico in Bari, Italy. The study was carried out in adherence to Good Clinical Practice, ICH Harmonized Tripartite Guidelines for Good Clinical Practice, and Declaration of Helsinki, and was approved by the local Institutional Ethics Committee (study no. 7777, approved on 31 May 2023) (NCT06120556). This study was conducted in accordance with the STROBE (Strengthening the Reporting of Observational Studies in Epidemiology) guidelines to ensure transparency and completeness in reporting. All patients with a scheduled visit at the diabetes outpatient clinic between 01 January 2014 and 31 August 2024 were assessed for eligibility. Main inclusion criteria were: males or females; Caucasian (White European); diagnosis of T2D within 5 years prior to enrolment; age at T2D onset >50 years; patients who were started on GLP‐1RA; availability of at least one follow‐up visit to our outpatient clinic between 6 and 12 months following GLP‐1RA initiation.

Main exclusion criteria were: diagnosis of autoimmune diabetes; monogenic diabetes; secondary diabetes; previous episodes of diabetic ketoacidosis (DKA); GLP‐1RA use prior to the index date; history of pancreatitis; lack of data useful for phenotype assignment (i.e., BMI, waist circumference, HbA1c, blood lipids); non‐adherence to GLP‐1RA prescription; lack of follow‐up visits.

Index date was set as the date patients were prescribed a GLP‐1RA.

For all the enrolled patients, the following data were extracted from clinical charts with reference to the time of GLP‐1 RA initiation: age, sex, age at diagnosis, duration of disease, BW, height, waist circumference, BMI, fasting blood glucose (FBG), HbA1c, lipid profile (total cholesterol, high‐density lipoprotein (HDL) and low‐density lipoprotein (LDL) cholesterol, triglycerides (TG)), creatinine, microalbuminuria, anti‐diabetic medications, diabetes complications (coronary artery disease (CAD) or cerebrovascular disease, retinopathy, peripheral neuropathy, nephropathy, peripheral artery disease (PAD), hepatic steatosis, heart failure (HF), chronic kidney disease defined as eGFR <60 mL/min/m^2^ (CKD)). Estimated glomerular filtration (eGFR) was calculated according to the CKD‐EPI equation.^12^

Included patients were assigned to the SIDD, SIRD, MOD and MARD phenotypes described by Ahlqvist et al. on the basis of a simplified calculation algorithm validated by Bello‐Chavolla and colleagues^13^ and available online (https://uiem.shinyapps.io/diabetes_clusters_app/). This model uses a machine‐learning self‐normalizing neural network (SNNN) approach trained on the NHANES database and requiring age, gender, age at diabetes onset, height, BMI, waist circumference, HbA1c, FBG, fasting TG and HDL cholesterol.^13^ In the absence of C‐peptide and insulin measurements, this algorithm exploits the metabolic score for insulin resistance (METS‐IR), which has been proven to be a reliable predictor of visceral adiposity and T2D.^14^ The equation to calculate the METS‐IR is (Ln ([2 × FBG] + [TG0] × BMI)/(Ln /HDL‐c)), and values above 51.13 have demonstrated an 85.1% sensitivity and 75.2% specificity in identifying insulin resistance.^14^ The algorithm by Bello‐Chavolla also exploits the METS‐VF index to estimate the amount of visceral adipose tissue, which has been consistently associated with metabolic and CV diseases.^14^, ^15^ METS‐VF is calculated as 4.466 + 0.011 × (Ln(METS‐IR))3 + 3.239 × (Ln(WHtr))3 + 0.319*(gender) + 0.594*(Ln(age)), where WHtr stands for waist‐to‐height ratio, and values above 7.18 have shown a 100% sensitivity and an 87.2% specificity in identifying patients with increased visceral adipose tissue.^15^

At the follow‐up visits, anthropometric (BW, BMI) and laboratory (HbA1c, FBG) features of glucose control were extracted from clinical charts.

The primary endpoint of the study was the difference in HbA1c change from baseline (%) at first follow‐up visit across the four prespecified diabetes phenotypes. Secondary endpoints were differences in percentages of patients (%) achieving or maintaining an HbA1c ≤7%, and in FBG (mg/dL) and BW (kg) changes from baseline among the four prespecified diabetes phenotypes. Which patients' characteristics could predict HbA1c reduction were also investigated. Additionally, differences in time to treatment failure, defined as the finding of an HbA1c level >7%, were investigated in the subgroup of patients from each phenotype who achieved an HbA1c target of ≤7% at first follow‐up visit.

According to the sample size calculation performed for the primary endpoint, evaluated with one‐way ANOVA, assuming a type I error of 0.05, power of 0.80 and a mean estimated effect size of 0.3, the enrolment of at least 126 patients was required. Moreover, an additional power analysis was performed to ensure appropriate sample size for the multivariable linear regression model assessing which patients' characteristics could predict HbA1c reduction. According to the commonly cited empirical rule for linear regression (*n* ≥ 50 + 8 × *k*, where *k* is the number of predictors), a sample size of 74 would suffice for models including up to three independent variables. Furthermore, a formal power calculation for a linear multiple regression (fixed model, R^2^ deviation from zero) with a medium effect size (*f* ^2^ = 0.15), α = 0.05, power = 0.80, and three predictors yielded a required sample size of 77 patients.

The statistical analysis plan was structured in a stepwise approach to first describe observed phenotype‐related differences, then explore which baseline features were most predictive of treatment response, and finally confirm those associations in adjusted models.

Normally distributed continuous variables are reported as mean (standard deviation), while non‐normally distributed variables are reported as median (interquartile range, IQR), and categorical variables are presented as counts (percentage). A two‐tailed paired Student t test was performed for normally distributed continuous variables, and a chi‐squared test was used to test for differences in categorical variables. Differences among prespecified phenotypes were assessed with one‐way ANOVA for normally distributed variables (Levene test was used to assess the homogeneity of variances and Welch correction was applied for *p* < 0.05).

Univariate regression was used to determine the association of baseline HbA1c with HbA1c change from baseline in each of the prespecified diabetes phenotypes after GLP‐1RA treatment. SHAP (SHapley Additive exPlanations) analysis, grounded in cooperative game theory, was used to identify and rank features that might predict response to GLP‐1RA in a model‐agnostic and non‐parametric setting. SHAP analysis allows us to quantify the importance of each variable in HbA1c change from baseline prediction and describes the effect of each variable on the outcome as depicted in SHAP summary and dependence plots.^16^ Variables with more than 30% missing values were excluded from SHAP analysis. The top‐ranked variables from this analysis were then included in a multivariable linear regression model to quantify their independent associations with treatment response in an interpretable and adjusted framework. To further test the potential mediating role of a specific variable, a causal mediation analysis using the mediation R package was performed to formally quantify the mediated and direct effects. Kaplan‐Meier survival analysis with log‐rank test and Cox regression were used to assess time to treatment failure according to diabetes phenotypes. Statistical analysis was performed with G*Power (v 3.1), JASP (v. 0.19.1 for Mac OS Apple Silicon) and RStudio (v. 2024.12.0 + 467).

## 3 RESULTS

Of the 6574 patients assessed, a total of 181 met the inclusion criteria and were enrolled in the study (Figure S1). Baseline features of the study population are summarized in Table 1.

**TABLE 1:** Baseline characteristics of the study population.

	Total	MARD	MOD	SIDD	SIRD	*p*
*N*	181	64 (35.4%)	33 (18.2%)	30 (16.6%)	54 (29.8%)	
Females, *N*	52 (35.9%)	15 (23.4%)	14 (42.4%)	10 (33.3%)	26 (48.1%)	**0.04**
Age, years	63.5 (7.5)	68.3 (7.1)	55.7 (3.3)	59.9 (5.7)	64.5 (5.9)	**<0.001**
Age at diagnosis, years	61.0 (7.3)	65.2 (7.0)	53.3 (2.5)	58.1 (5.9)	62.5 (5.8)	**<0.001**
Diabetes duration, years	2.4 (1.9)	3.1 (1.9)	2.4 (1.9)	1.8 (1.8)	2.0 (1.8)	**<0.01**
BW, kg	91.4 (18.1)	79.3 (11.8)	105.5 (19.5)	89.5 (13.0)	98.2 (16.6)	**<0.001**
BMI, kg/m^2^	33.1 (5.7)	28.4 (2.6)	38.0 (5.9)	31.5 (3.4)	36.6 (4.5)	**<0.001**
Waist circumference, cm	111.7 (13.0)	103.6 (9.6)	120.9 (14.3)	109.3 (11.1)	117.2 (10.6)	**<0.001**
FBG, mg/dL	148.3 (43.8)	141.3 (34.1)	150.6 (30.4)	203.1 (55.2)	124.7 (23.9)	**<0.001**
HbA1c, %	7.3 (1.3)	7.0 (0.8)	7.2 (0.7)	9.4 (1.7)	6.7 (0.7)	**<0.001**
Total cholesterol, mg/dL	167.6 (41.3)	152.8 (37.9)	179.6 (44.8)	185.9 (40.8)	167.6 37.7)	**<0.001**
HDL cholesterol, mg/dL	47.5 (12.6)	46.9 (11.4)	45.7 (9.5)	44.9 (8.9)	50.6 (16.4)	0.12
TG, mg/dL	154.8 (87)	143.4 (82.1)	160.6 (96.3)	201 (116.7)	139.2 (55.2)	**0.009**
LDL cholesterol, mg/dL	89 (34.4)	77.2 (31.9)	101.8 (34.6)	100.8 (36.0)	89.3 (31.9)	**0.001**
eGFR, mL/min/1.73 m^2^	82.2 (17.7)	75.5 (17.7)	90.2 (16.4)	90.4 (15.6)	80.8 (16.2)	**<0.001**
Diabetes complications						
CAD, *N* (%)	40 (22.1)	22 (28)	6 (18.2)	2 (6.6)	10 (18.5)	**0.01**
Cerebrovascular disease, *N* (%)	9 (5)	6 (9.4)	1 (3)	0 (0)	2 (3.7)	0.20
Carotid plaque, *N* (%)	94 (51.9)	38 (59.4)	14 (42.4)	18 (60.0)	24 (44.4)	0.20
HF, *N* (%)	7 (3.9)	1 (1.6)	1 (3.0)	1 (3.3)	4 (7.4)	0.42
Diabetic neuropathy, *N* (%)	21 (11.6)	5 (7.8)	6 (18.2)	4 (13.3)	6 (11.1)	0.50
Diabetic retinopathy, *N* (%)	12 (6.7)	6 (9.5)	2 (6.1)	3 (10)	1 (1.8)	0.33
CKD, *N* (%)	23 (12.7)	12 (18.7)	1 (3.0)	2 (6.6)	8 (14.8)	**0.03**
DFU, *N* (%)	5 (3.0)	0 (0)	3 (9.7)	0 (0)	2 (4.1)	0.05
Background antidiabetes medications						
Metformin, *N* (%)	142 (78.5)	52 (81.5)	28 (84.8)	23 (72.2)	39 (72.2)	0.49
Pioglitazone, *N* (%)	2 (1.1)	1 (1.6)	0 (0)	1 (3.3)	0 (0)	0.48
DPP‐4i, *N* (%)	22 (12.2)	12 (18.7)	2 (6.1)	2 (6.6)	6 (11.1)	0.20
SGLT‐2i, *N* (%)	24 (13.3)	12 (18.7)	6 (18.2)	3 (10)	3 (5.5)	0.14
Glinides/SU, *N* (%)	10 (5.5)	3 (4.7)	1 (3.0)	1 (3.3)	5 (9.6)	0.53
Insulin, *N* (%)	21 (11.6)	7 (10.9)	3 (9.1)	7 (23.3)	4 (7.4)	0.16

*Note*: *P*‐value refers to differences among MARD, MOD, SIDD and SIRD phenotypes. Normally distributed continuous variables are reported as mean (standard deviation) and categorical variables are presented as counts (percentage). Statistically significant results are presented in bold.

Abbreviations: BMI, body mass index; BW, body weight; CAD, coronary artery disease; DFU, diabetic foot ulcer; DPP‐4i, dipeptidyl peptidase‐4 inhibitors; FBG, fasting blood glucose; IQR, interquartile range; MARD, mild age‐related diabetes; MOD, mild obesity‐related diabetes; *N*, number; SD, standard deviation; SGLT‐2i, sodium‐glucose cotransporter 2 inhibitors; SIDD, severe insulin‐deficient diabetes; SIRD, severe insulin‐resistant diabetes; SU, sulfonylureas; TG, triglycerides.

Included patients were in the majority males (64.1%), with a mean age of 63.5 (7.5) years, age at diagnosis of 61 (7.3) years, and diabetes duration of 2.4 (1.9) years. The mean BMI was 33.1 (5.7) kg/m^2^ and mean waist circumference was 111.7 (13) cm; glycaemic control was suboptimal, with mean HbA1c of 7.3 (1.3) % and mean FBG of 148.3 (43.8) mg/dl. Mean eGFR was 82.2 (17.7) mL/min/1.73m^2^, and 12.7% patients had CKD. The most represented phenotype was MARD (35.4%, 64 patients), followed by SIRD (29.8%, 54 patients), MOD (18.2%, 33 patients) and SIDD (16.6%, 30 patients).

As expected, the four prespecified phenotypes differed in most patients' characteristics, except for HDL cholesterol. For instance, MOD patients were the youngest, MOD and SIRD patients showed the greatest baseline BW, BMI and waist circumference, while SIDD patients displayed the highest HbA1c and FBG levels. Indeed, mean baseline HbA1c was significantly higher in the SIDD compared with the other phenotypes (9.4 [1.7] %, vs. 7.0 [0.8] % in MARD, 7.2 [0.7] % in MOD, and 6.7 [0.7] % in SIRD, *p* < 0.001).

No differences in background baseline anti‐diabetes therapy and diabetes complications were found among the four phenotypes, except for a greater prevalence of CAD in MARD and CKD in MARD and SIRD patients. The majority of patients were started on dulaglutide (49.2%), followed by oral semaglutide (19.3%), once‐weekly subcutaneous semaglutide (17.1%), liraglutide (12.1%) and once‐weekly exenatide (2.8%), without differences among the four phenotypes (Table S2). All considered baseline features had no missing values, except for albuminuria.

**FIGURE 1:**
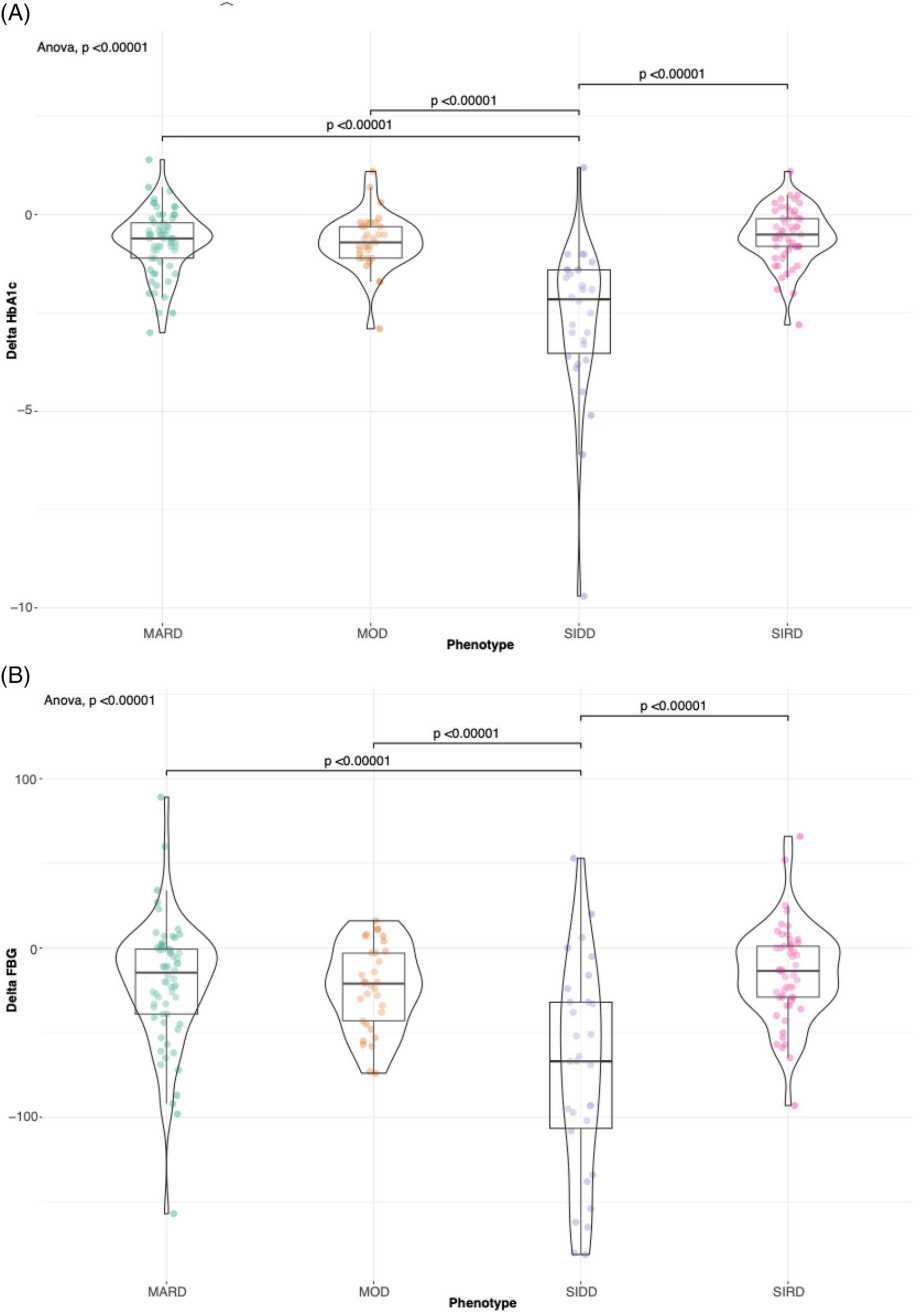
Change from baseline in HbA1c (A) and FBG (B) according to diabetes phenotype. MARD, mild age‐related diabetes; MOD, mild obesity‐related diabetes; SIDD, severe insulin‐deficient diabetes; SIRD, severe insulin‐resistant diabetes.

The first follow‐up visit occurred after a median of 8.4 (2.3) months. HbA1c, FBG and BW were significantly reduced across all the phenotypes following GLP‐1RA initiation (Table 2). Mean HbA1c and FBG reduction were significantly greater in patients belonging to the SIDD phenotype versus all other phenotypes (−2.6 [1.9] % vs. −0.7 [0.8] % in MARD, −0.7 [0.7] % in MOD, −0.5 [0.7] % in SIRD individuals, *p* < 0.0001, and − 72.4 [37.2] mg/dL vs. −20.6 [37.2] mg/dL in MARD, −23.4 [25.2] mg/dL in MOD, −14.4 [27.5] mg/dL in SIRD individuals, *p* < 0.0001, respectively) (Figure 1A,B). At the first follow‐up visit, 146 out of 181 patients achieved or maintained the HbA1c target of ≤7%; a similar percentage of patients achieving or maintaining HbA1c ≤7% was detected across phenotypes (72.7–90.7%, *p* = 0.1). Notably, change from baseline in BW did not differ among phenotypes (Figure S2). The four phenotypes differed significantly in several baseline clinical characteristics (Table 1), which could have contributed to the differences observed in HbA1c reduction. To address these potential confounding factors, we first applied SHAP analysis to identify the most influential baseline variables associated with glycaemic response. These variables were then included in a multivariable linear regression model, allowing us to adjust for relevant clinical covariates and better isolate the independent effect of diabetes phenotype. The SHAP analysis confirmed baseline HbA1c as the most important feature for HbA1c change from baseline prediction in our cohort, with a mean SHAP value of 0.657, followed by baseline body weight and HDL cholesterol (mean SHAP value of 0.070 and 0.046, respectively) by a substantial margin (Figure S2A). Of note, assigned phenotype ranked much lower in importance (SHAP value 0.00). As depicted in the dependence graph, greater values of baseline HbA1c led to a greater HbA1c reduction (Figure S2B).

**TABLE 2:** Change from baseline in HbA1c, FBG, and BW according to phenotype.

	Total	MARD	MOD	SIDD	SIRD
HbA1c, %	−0.9 (1.3)	−0.7 (0.8)	−0.7 (0.7)	−2.6 (1.9)	−0.5 (0.7)
*p*	<0.001	<0.001	<0.001	<0.001	<0.001
FBG, mg/dl	−27.9 (43.0)	−20.6 (37.2)	−23.4 (25.2)	−72.4 (62.0)	‐14.4 (27.5)
*p*	<0.001	<0.001	<0.001	<0.001	<0.001
BW, kg	−2.8 (6.2)	−1.6 (6.1)	−4.4 (7.4)	−3.9 (6.4)	−2.6 (5.2)
*p*	<0.001	<0.001	<0.001	<0.001	<0.001

*Note*: Continuous variables are presented as mean (standard deviation). *P*‐value refers to change from baseline.

Abbreviations: BW, body weight; FBG, fasting blood glucose; MARD, mild age‐related diabetes; MOD, mild obesity‐related diabetes; OW, once weekly; SIDD, severe insulin‐deficient diabetes; SIRD, severe insulin‐resistant diabetes.

**FIGURE 2:**
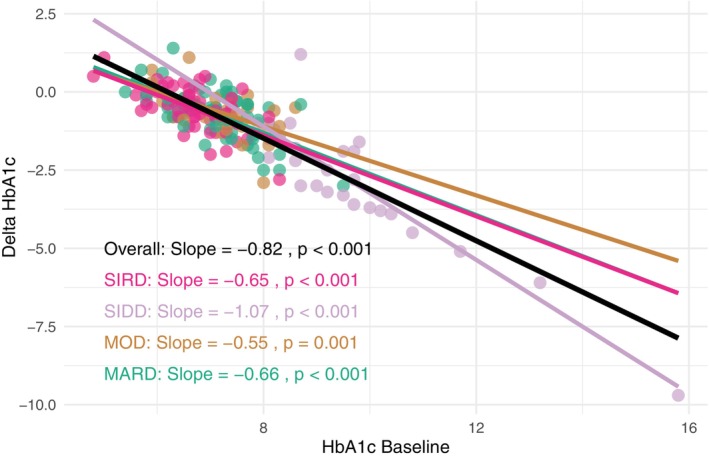
Baseline HbA1c as a predictor of HbA1c reduction in the overall cohort and across phenotypes. MARD, mild age‐related diabetes; MOD, mild obesity‐related diabetes; SIDD, severe insulin‐deficient diabetes; SIRD, severe insulin‐resistant diabetes.

The top 10 features resulting from the SHAP analysis, namely baseline HbA1c, FBG, BW, creatinine, LDL, HDL and triglycerides, age, gender and diabetes duration, were considered for a stepwise multivariable regression analysis (Table S2). As expected, baseline HbA1c had a predominant role in determining change in HbA1c; the negative coefficient (β = −0.833, *p* < 0.001) indicated that for each unit increase in baseline HbA1c, an approximate reduction of 0.833 units in HbA1c variation is expected. In addition, male gender and longer diabetes duration were significantly associated with marginally relevant changes in HbA1c. Consistently, baseline HbA1c was significantly associated with HbA1c reduction, both in the overall population (slope = −0.82, 95% CI −0.89, −0.75; *p* < 0.001) and each phenotype (Figure 2). Confirming that the association between phenotypes and HbA1c reduction was mediated by baseline HbA1c, a causal mediation analysis showed that the effect of the SIDD phenotype on HbA1c reduction was entirely mediated by baseline HbA1c levels (*p* < 0.001), while the direct effect of the phenotype was not statistically significant (ADE =0.007, *p* = 0.86) (Table S3).

Accordingly, baseline FBG and diabetes duration were significantly associated with change in FBG following GLP‐1RA initiation (Table S4, Figure S3), while baseline BW showed a significant association with change in BW although the overall explanatory power of the regression model remained limited (Table S5, Figure S4).

**FIGURE 3:**
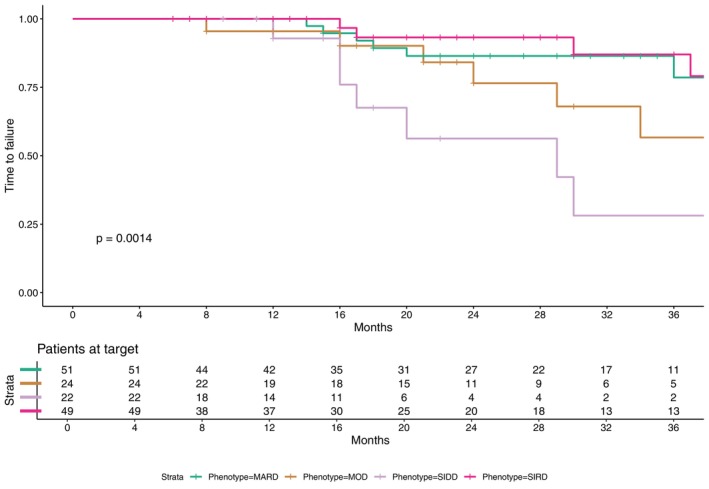
Time to treatment failure according to diabetes phenotypes. Kaplan–Meier survival curves illustrating time to treatment failure over a follow‐up period of up to 36 months, stratified by diabetes phenotype. The p‐value was calculated using the log‐rank test. MARD, mild age‐related diabetes; MOD, mild obesity‐related diabetes; SIDD, severe insulin‐deficient diabetes; SIRD, severe insulin‐resistant diabetes.

Finally, time to failure was significantly different among diabetes phenotypes (unadjusted HR with respect to MARD: 1.72 (0.65–4.57) in MOD, 3.68 (1.37–9.94) in SIDD, 0.38 (0.12–1.27) in SIRD, log‐rank test *p* < 0.01) (Figure 3); specifically, belonging to the SIDD phenotype was associated with an increased risk of earlier treatment failure as shown in the unadjusted Cox proportional hazard model (Table 3, Model 1). Importantly, to better understand whether this association was driven by intrinsic characteristics of the phenotype or by confounding clinical variables, the model was extended to include baseline HbA1c, gender and diabetes duration. This adjustment improved the performance of the model (concordance increased from 0.68 to 0.72); however, the association between the SIDD phenotype and time to failure was no longer statistically significant (HR 2.63, 95% CI 0.67–10.36) (Table 3, Model 2).

**TABLE 3:** Cox proportional hazards models for survival analysis.

	HR	95% CI	*p*
Model 1			
MOD	1.72	0.65–4.57	0.27
SIDD	3.68	1.37–9.94	**<0.01**
SIRD	0.38	0.12–1.27	0.12
Concordance: 0.68 Likelihood ratio test = 13.39 on 3 df, *p* = 0.004; Wald test = 12.92 on 3 df, *p* = 0.005 Score (logrank) test = 15.81 on 3 df, *p* = 0.001; Proportionality of hazards test using Schoenfeld residuals, *p* = 0.51			
Model 2			
MOD	2.01	0.70–5.78	0.19
SIDD	2.63	0.67–10.36	0.16
SIRD	0.59	0.17–2.10	0.41
Baseline HbA1c	1.36	0.94–1.95	0.09
DM duration	1.16	0.92–1.46	0.21
Gender (male)	0.84	0.46–1.53	0.57
Concordance: 0.72 Likelihood ratio test = 17.98 on 6 df, *p* = 0.006; Wald test = 17.83 on 6 df, *p* = 0.007 Score (logrank) test = 20.99 on 6 df, *p* = 0.0002; Proportionality of hazards test using Schoenfeld residuals, *p* = 0.68 (global)		

*Note*: HR for diabetes phenotypes is expressed with respect to MARD.

Abbreviations: MOD, mild obesity‐related diabetes; SIDD, severe insulin‐deficient diabetes; SIRD, severe insulin‐resistant diabetes.

## 4 DISCUSSION

Our results showed that SIDD patients exhibited a greater HbA1c reduction with GLP1‐RA compared with those assigned to the other phenotypes. However, baseline HbA1c, rather than assignment to SIDD phenotype per se, was the most relevant predictor of HbA1c reduction, as SIDD patients displayed the greatest baseline values. Our results are in line with those of the recent trial conducted by Dwibedi et al., showing that baseline HbA1c and HOMA‐2B values, indicating poor insulin secretion, may better explain HbA1c variation compared with phenotype assignment.^11^ Interestingly, in that study, response to semaglutide was more strongly related to baseline HbA1c than response to dapagliflozin.^11^ Accordingly, several studies with different designs showed the prominent role of baseline HbA1c in predicting glucose‐lowering efficacy of GLP‐1RA, including some RCT,^17^, ^18^ a pooled post‐hoc analysis of prospective Semaglutide Real‐world Evidence (SURE) studies on 1212 patients,^19^ and several retrospective studies.^20^, ^21^, ^22^ However, it should be noted that patients classified as SIDD are characterized by marked insulin deficiency and typically present with higher glycaemic levels at diagnosis, which likely accounts for the greater baseline HbA1c values observed in this group.

In our cohort, diabetes duration and gender were also associated with HbA1c reduction, although to a lesser extent. Accordingly, several retrospective studies also found that individuals with a shorter diabetes duration were more likely to achieve greater glucose control.^20^, ^21^, ^22^, ^23^ Moreover, in a cohort of 620 individuals with T2D initiating GLP‐1RA, longer diabetes duration and lower C‐peptide levels were linked to reduced glucose lowering effectiveness.^24^ The impact of gender in GLP‐1RA‐mediated glucose reduction is still debated^6^, ^17^, ^18^ and, given the minimal effect observed, further investigation is warranted to determine whether our findings are significant or potentially attributable to chance.

Furthermore, SHAP analysis identified baseline BW, creatinine and cholesterol levels as potential predictors of HbA1c lowering. An analysis of genome‐wide association studies (GWAS) identified five robust clusters of genetic variants and traits differing in BW and lipid profiles, highlighting the relevance of these features as markers of heterogeneity in the pathogenesis of T2D.^25^ However, we could not identify a clear pattern in the association between these variables and HbA1c reduction following GLP‐1RA initiation, similarly to the study by Dwibedi et al.^11^

Soft clustering approaches, where patients may have features belonging to more than one of the predefined subtypes, or regression models, integrating clinical variables to prompt clinical decisions for patients falling above a specific threshold, have been proposed as an alternative to hard clustering.^8^ Dennis et al. showed that the hard clustering approach proposed by Ahlqvist et al. was outperformed by models using simple clinical features for treatment selection and prediction of metformin, sulfonylureas and thiazolidinediones' glucose‐lowering effects in patients enrolled in the ADOPT and RECORD trials.^9^ Moreover, as patients' features change with time due to the effect of treatment and/or disease progression, phenotype assignment might change during follow‐up, as shown by Zaharia et al., demonstrating a switch in 23% of patients at 5 years.^26^ In line with these considerations, Cardoso et al. developed and validated a novel algorithm to predict differences in response to SGLT‐2i and GLP‐1RA at one year, based on routinely available clinical variables.^6^

To the best of our knowledge, we are the first to investigate time‐to‐treatment failure according to diabetes phenotypes as defined by Ahlqvist and colleagues. Interestingly, we found that patients belonging to the SIDD phenotype also experienced an earlier treatment failure versus other phenotypes. However, assignment to a specific phenotype lost significance as a predictor of time to treatment failure when baseline HbA1c, diabetes duration and gender were added to the model.

Nair et al. also exploited the ADOPT dataset but used a DDRTree algorithm creating a two‐dimensional non‐linear tree structure with the distal ends of the tree branches depicting extreme and the tree centre mixed phenotypes.^27^ These Authors demonstrated that failure of monotherapy with metformin or sulfonylureas occurred faster in obese and hyperglycaemic individuals, while failure of monotherapy with thiazolidinediones was faster in those with lipodystrophy‐related genes and low risk of obesity.^27^ Predictors of time‐to‐treatment failure have not been extensively studied, and it could be useful to know which patients progress faster as they will need prompt therapy intensification to minimize clinical inertia.

Although well aligned with everyday clinical practice, the retrospective and pragmatic design of our study comes with several limitations. Firstly, we were unable to assess the role of beta‐cell function in response to treatment as C‐peptide and fasting insulin measurements are not part of routine clinical practice. Also, due to the lack of GADA, C‐peptide, and insulin measurements in our dataset, we used the simplified and validated classification algorithm developed by Bello‐Chavolla et al., which was specifically designed to identify the Ahlqvist phenotypes in real‐world settings using only routinely collected clinical and biochemical variables. Of note, poor insulin secretion was identified as a predictor of greater HbA1c reduction following semaglutide administration.^11^ Likewise, since pancreatic autoimmunity is not routinely evaluated in adult patients without risk factors for autoimmune diabetes, GAD antibodies measurement was not available for most patients in our cohort. Hence, we attempted to avoid the inclusion of SAID patients by enrolling individuals aged >50 years at T2D diagnosis and excluding those with known autoimmune diabetes or previous DKA. Also, we were unable to assess whether treatment with GLP‐1RA differentially reduced CV and renal events according to patients' phenotypes due to unstandardized and suboptimal reporting of these complications. Finally, despite being a single‐centre study, our findings are strongly aligned with those of a retrospective analysis of 4467 patients with T2D from the Diabetes Patients Follow‐up registry, which identified higher baseline HbA1c and male gender as predictors of greater HbA1c reduction, while associating longer diabetes duration with smaller improvements in glycaemic control.^28^

In summary, our study demonstrates that GLP‐1RA induced greater HbA1c reduction in the SIDD phenotype. However, baseline HbA1c, and to a lesser extent gender and diabetes duration, rather than phenotype assignment per se, predicted HbA1c reduction following GLP‐1RA initiation. Moreover, SIDD patients showed earlier treatment failure (HR 3.69, 95% CI 1.37–9.94; *p* < 0.01), but this effect also disappeared after adjusting for baseline HbA1c. These findings emphasize the central role of routinely available clinical variables (e.g., baseline glycaemic control) in predicting both treatment response and durability of effectiveness, and suggest that hard clustering approaches to T2D phenotyping may have limited added value in guiding GLP‐1RA prescription. Future prospective studies, ideally incorporating measures of beta‐cell function and pancreatic autoimmunity, are needed to validate these findings and to refine stratification tools for personalized diabetes care.

## AUTHOR CONTRIBUTIONS

Conceptualization: IC, FGiordano, AC, GPS, FGiorgino; Data curation: IC, FGiordano, IIM; Methodology and formal analysis: IC, LDG; Supervision: AC, AN, GPS, LL, FGiorgino; Visualization: IC, LDG; Writing (original draft): IC; Writing (review and editing): IC, SDM, AC, GPS, FGiorgino.

## FUNDING INFORMATION

Next Generation EU, in the context of the National Recovery and Resilience Plan, Investment PE8 – Project Age‐It: ‘Ageing Well in an Ageing Society’. This resource was co‐financed by the Next Generation EU [DM 1557 11.10.2022]. The views and opinions expressed are only those of the authors and do not necessarily reflect those of the European Union or the European Commission; neither the European Union nor the European Commission can be held responsible for them.

## CONFLICT OF INTEREST STATEMENT

Angelo Cignarelli has received speakers honoraria from AstraZeneca, Eli Lilly, Novo Nordisk, Roche Diagnostics and Sanofi. Fiorella Giordano received speakers honoraria from Eli Lilly and Novo Nordisk. Francesco Giorgino has been on advisory boards, received consulting fees or speaker honoraria from AstraZeneca, Boehringer‐Ingelheim, Eli Lilly, Lifescan, Medimmune, Merck Sharp & Dohme, Medtronic, Novo Nordisk, Roche Diabetes Care, Sanofi. Gian Pio Sorice has been on advisory boards, received consulting fees or speaker honoraria from Amgen, Amryt Pharma, Eli Lilly, Farmitalia, Novo Nordisk, Sanofi; Irene Caruso has received speaker fees or travel grants from Abbott, Guidotti SpA, Eli Lilly; Ludovico Di Gioia has been on advisory boards, received consulting fees or speaker honoraria from Abbott, Eli Lilly, Lusofarmaco, MOVI SpA, Novo Nordisk, Roche Diabetes Care, Sanofi, Theras. Luigi Laviola has been on advisory boards, received consulting fees or speaker honoraria from Abbott, AstraZeneca, Boehringer‐Ingelheim, Eli Lilly, Merck Sharp & Dohme, Medtronic, Menarini, MOVI SpA, Mundipharma, Novo Nordisk, Roche Diabetes Care, Sanofi, Terumo. Sergio Di Molfetta has received speaker fees from Ascensia Diabetes Care, MOVI SpA, Roche Diabetes Care.

## PEER REVIEW

The peer review history for this article is available at https://www.webofscience.com/api/gateway/wos/peer-review/10.1111/dom.70005.

## Supporting information


**Table S1.** GLP‐1RA prescribed to included patients.
**Table S2.** Stepwise multivariate regression model for prediction of HbA1c change from baseline.
**Table S3.** Causal mediation analysis to assess the role of baseline HbA1c as a mediator of the effect of T2D phenotypes on HbA1c reduction.
**Table S4.** Stepwise multivariate regression model for prediction of FBG change from baseline.
**Table S5.** Stepwise multivariate regression model for prediction of BW change from baseline.
**Figure S1.** Patients’ disposition.
**Figure S2.** SHAP summary plot for the 10 most relevant features for change in HbA1c prediction (A) and dependence graph for HbA1c (B).
**Figure S3.** SHAP summary plot for change in fasting blood glucose (FBG).
**Figure S4.** SHAP summary plot for change in body weight (BW).

## Data Availability

The data supporting this study are not publicly available due to ethical and privacy restrictions, but can be accessed upon request from the corresponding author and approval from the appropriate ethics committee.
