# Supplementary material for: Effectiveness of GLP‐1RA according to different type 2 diabetes phenotypes: A retrospective study
Source: Diabetes Obes Metab. 2025 Aug 11;27(11):6210–9. doi: 10.1111/dom.70005 (PMC12515759; doi:10.1111/dom.70005)

**Supplementary appendix**

**Supplementary Table 1.** GLP-1RA prescribed to included patients.

|  | **Total** | **MARD** | **MOD** | **SIDD** | **SIRD** | ***p*** |
| --- | --- | --- | --- | --- | --- | --- |
| Liraglutide, N | 22 (12.1) | 8  (12.5) | 6  (18.2) | 3  (10.0) | 5  (9.2) | 0.64 |
| Exenatide OW, N | 5  (2.8) | 2  (3.1) | 3  (9.1) | 0  (0) | 0  (0) | 0.06 |
| Dulaglutide, N | 89  (49.2) | 39  (60.9) | 11  (33.3) | 13  (43.3) | 26  (48.1) | 0.06 |
| Semaglutide OW, N | 31  (17.1) | 7  (10.9) | 8  (24.2) | 5  (16.6) | 11  (20.4) | 0.35 |
| Semaglutide oral, N | 35  (19.3) | 9  (14.1) | 5  (15.1) | 9  (30) | 12  (22.2) | 0.26 |

MARD, mild age-related diabetes; MOD, mild obesity-related diabetes; OW, once weekly; SIDD, severe insulin-deficient diabetes; SIRD, severe insulin-resistant diabetes.

Categorical variables are presented as counts (percentage). p-value refers to differences among MARD, MOD, SIDD and SIRD phenotypes.

**Supplementary Table 2. Stepwise multivariate regression model for prediction of HbA1c change from baseline**

|  | **β** | **p** |
| --- | --- | --- |
| (Intercept) | 4.900 | <0.001 |
| HbA1c baseline | -0.833 | <0.001 |
| Gender (male) | -0.261 | <0.01 |
| DM duration | 0.106 | <0.01 |
| R^2^ = 0.754, Adjusted R^2^ = 0.750  AIC = 362.68, BIC = 378.68 | | |

The following covariates were considered but not included: LDL and HDL cholesterol, triglycerides, age, baseline creatinine and body weight. FBG baseline was excluded due to collinearity. Standardized coefficients were reported for continuous variables.

DM, diabetes mellitus.

## **Supplementary Table 3.** Causal mediation analysis to assess the role of baseline HbA1c as a mediator of the effect of T2D phenotypes on HbA1c reduction.

|  | **Estimate** | **95%CI** | **p** |  |
| --- | --- | --- | --- | --- |
| ACME | -2.04 | -2.86;-1.27 | <0.001 |  |
| ADE | 0.07 | -0.45;0.53 | 0.86 |  |
| Total effect | -1.97 | -2.77;-1.27 | <0.001 |  |
| Proportion mediated | 1.03 | 0.78;1.32 | <0.001 |  |
| Sample size used: 181  Simulations: 1000  ACME, Average Causal Mediation Effect; ADE, Average Direct Effect. | | | | |

## **Supplementary Table 4.** Stepwise multivariate regression model for prediction of FBG change from baseline.

|  | **β** | **p** |
| --- | --- | --- |
| (Intercept) | 70.28 | <0.001 |
| FBG baseline | -0.77 | <0.001 |
| DM duration | 2.64 | 0.01 |
| LDL cholesterol | 0.10 | 0.09 |
| SGLT2i | 10.03 | 0.09 |
| R^2^ = 0.6431 Adjusted R^2^ = 0.635, p-value <0.01 | | |

The top 10 variables resulting from SHAP analysis were used to build the stepwise multivariate regression model. Microalbuminuria and neuropathy were excluded due to a high number of missing data. Baseline BMI and height were excluded due to collinearity with baseline body weight, total cholesterol was excluded due to collinearity with LDL cholesterol and replaced by HDL cholesterol. The following covariates were considered but not included: age, baseline creatinine, baseline body weight, baseline HDL cholesterol and triglycerides, baseline HbA1c, baseline waist.

## **Supplementary Table 5.** Stepwise multivariate regression model for prediction of BW change from baseline.

|  | **β** | **p** |
| --- | --- | --- |
| (Intercept) | 5.89 | 0.01 |
| BW baseline | -0.10 | <0.001 |
| Full dose of GLP-1RA | 1.66 | 0.06 |
| R^2^ = 0.11 Adjusted R^2^ = 0.09, p-value <0.01 | | |

The top 10 variables resulting from SHAP analysis were used to build the stepwise multivariate regression model. Baseline BMI and height were excluded due to collinearity with baseline body weight, total cholesterol was excluded due to collinearity with LDL cholesterol and HDL cholesterol. The following covariates were considered but not included in the stepwise regression model: age, type of GLP-1RA, baseline LDL and HDL cholesterol and triglycerides, baseline HbA1c, and diabetes duration.

## **Supplementary Figure 1.** Patients’ disposition.


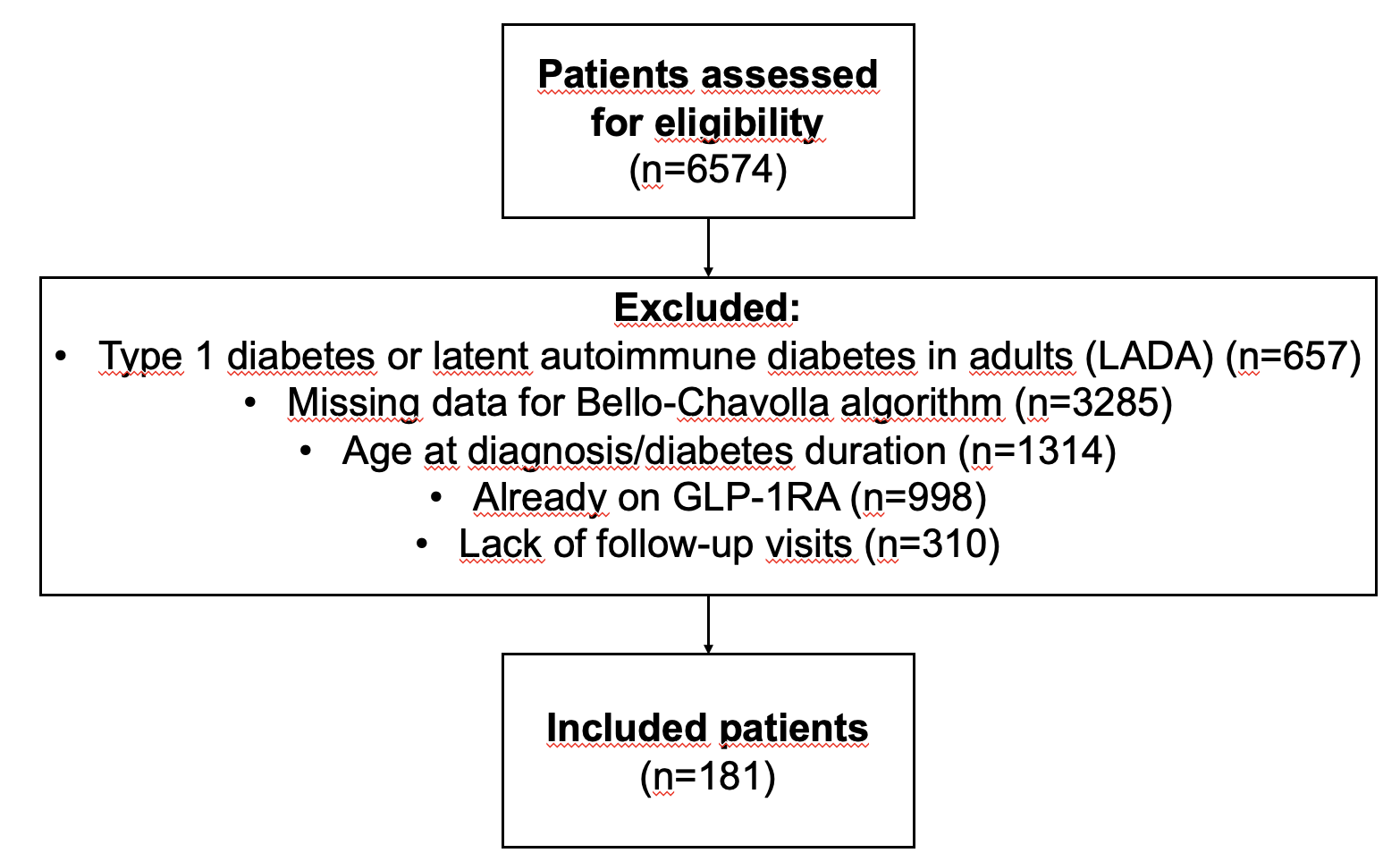


In the SHAP summary plot (A), the y-axis indicates predictors in order of importance from top to bottom, and the mean absolute SHAP value is reported besides each variable; the x-axis represents the SHAP values range. Purple and yellow colors indicate high and low values of each variable, respectively. For instance, high levels of baseline HbA1c, represented by purple dots, significantly reduce HbA1c, as shown by a lower SHAP value for this observation. Microalbuminuria was excluded from the analysis due to >30% missing values. Similarly, the dependence plot (B) displayed how baseline HbA1c impacted the model's prediction of HbA1c reduction across its different values.

BW, body weight; Cholesterol_tot, total cholesterol; DM, diabetes mellitus; FBG, fasting blood glucose.

**Supplementary Figure 3.** SHAP summary plot for change in fasting blood glucose (FBG)


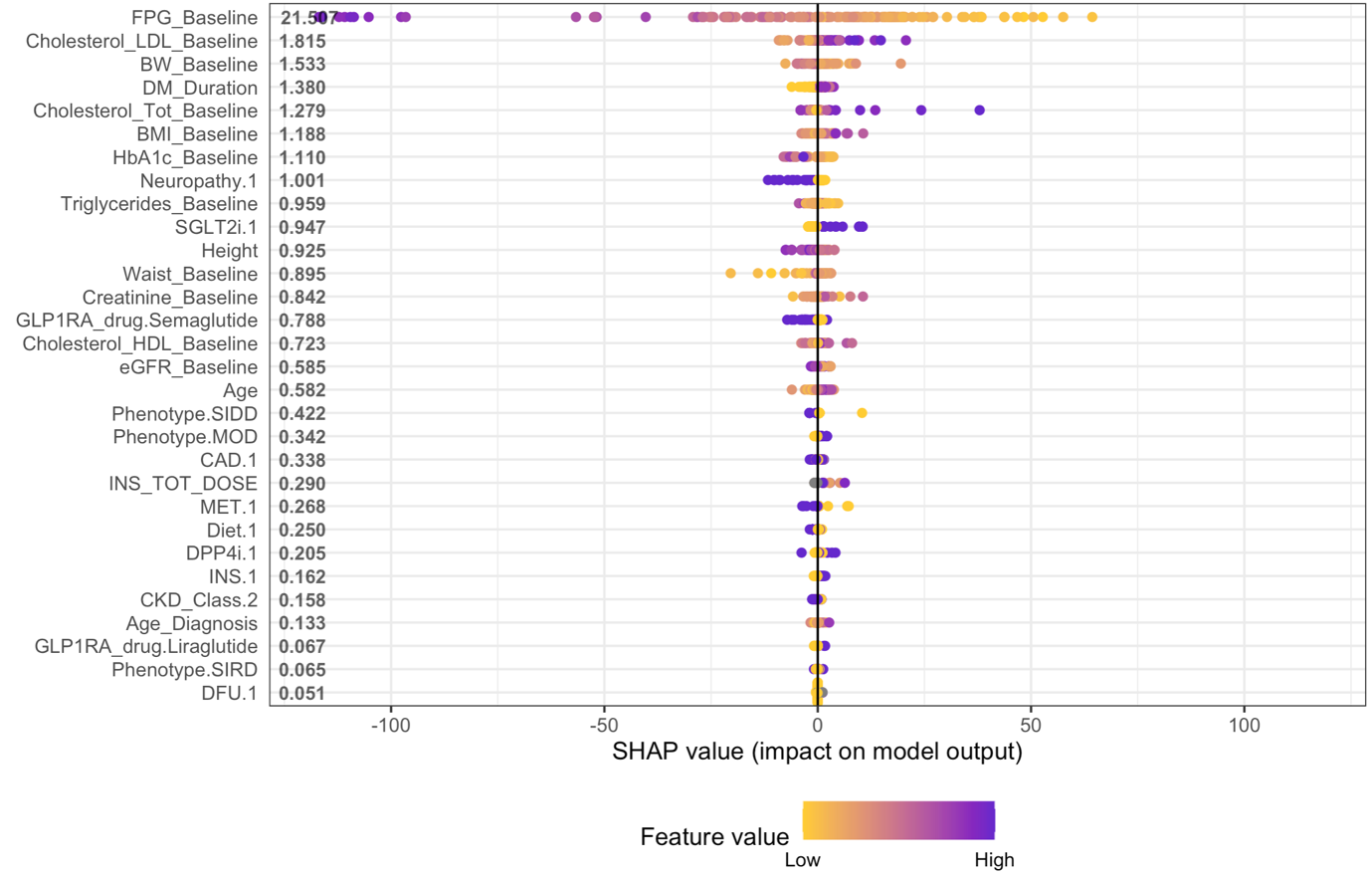


**Supplementary Figure 4**. SHAP summary plot for change in body weight (BW)


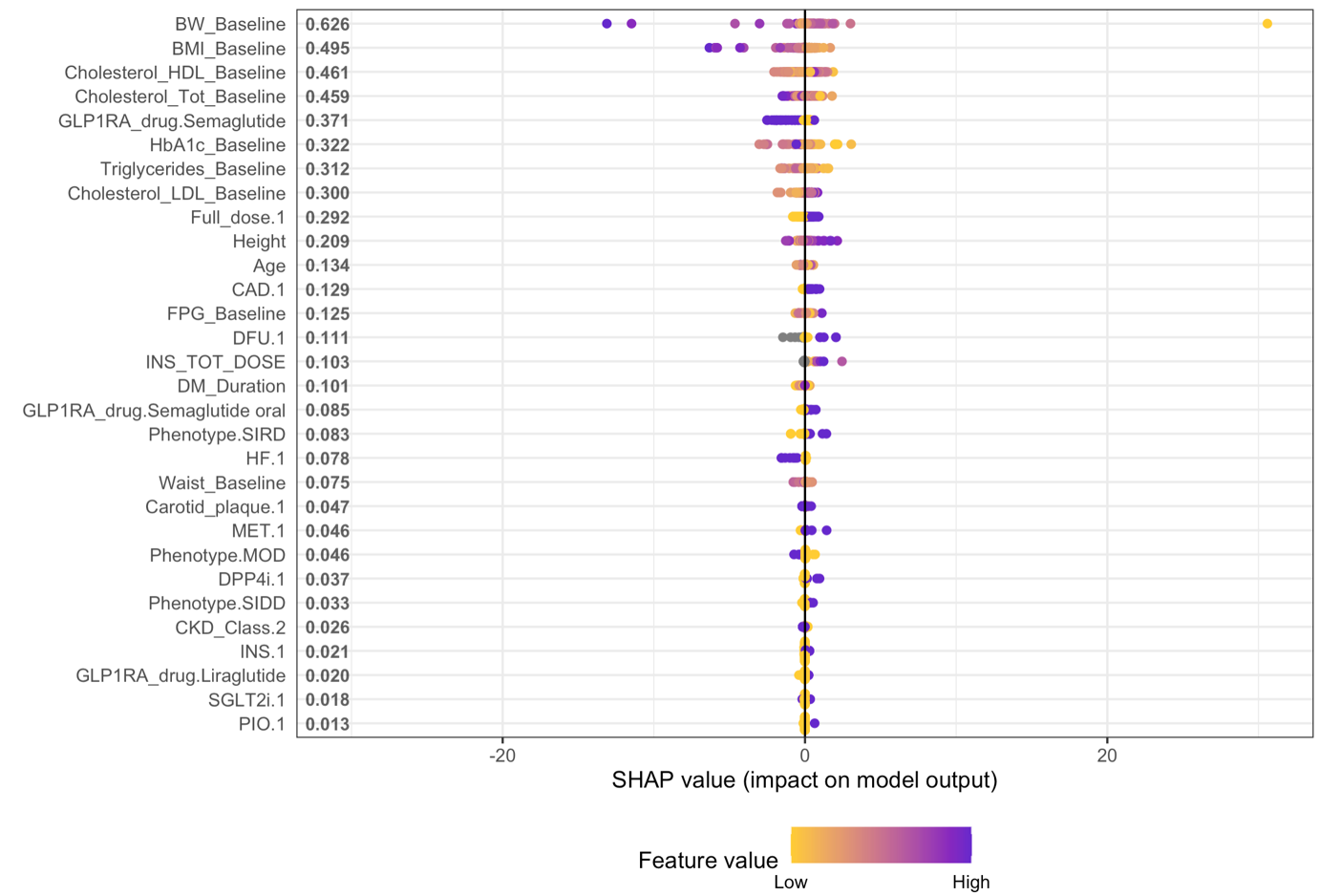

Supplement: Supplementary file 1 — Table S1. GLP‐1RA prescribed to included patients. Table S2. Stepwise multivariate regression model for prediction of HbA1c change from baseline. Table S3. Causal mediation analysis to assess the role of baseline HbA1c as a mediator of the effect of T2D phenotypes on HbA1c reduction. Table S4. Stepwise multivariate regression model for prediction of FBG change from baseline. Table S5. Stepwise multivariate regression model for prediction of BW change from baseline. Figure S1. Patients’ disposition. Figure S2. SHAP summary plot for the 10 most relevant features for change in HbA1c prediction (A) and dependence graph for HbA1c (B). Figure S3. SHAP summary plot for change in fasting blood glucose (FBG). Figure S4. SHAP summary plot for change in body weight (BW). [file DOM-27-6210-s001.zip › dom70005-sup-0001-Supinfo.docx]
