# Supplementary figures and images for: Effectiveness of GLP‐1RA according to different type 2 diabetes phenotypes: A retrospective study
Source: Diabetes Obes Metab. 2025 Aug 11;27(11):6210–9. doi: 10.1111/dom.70005 (PMC12515759; doi:10.1111/dom.70005)

A)

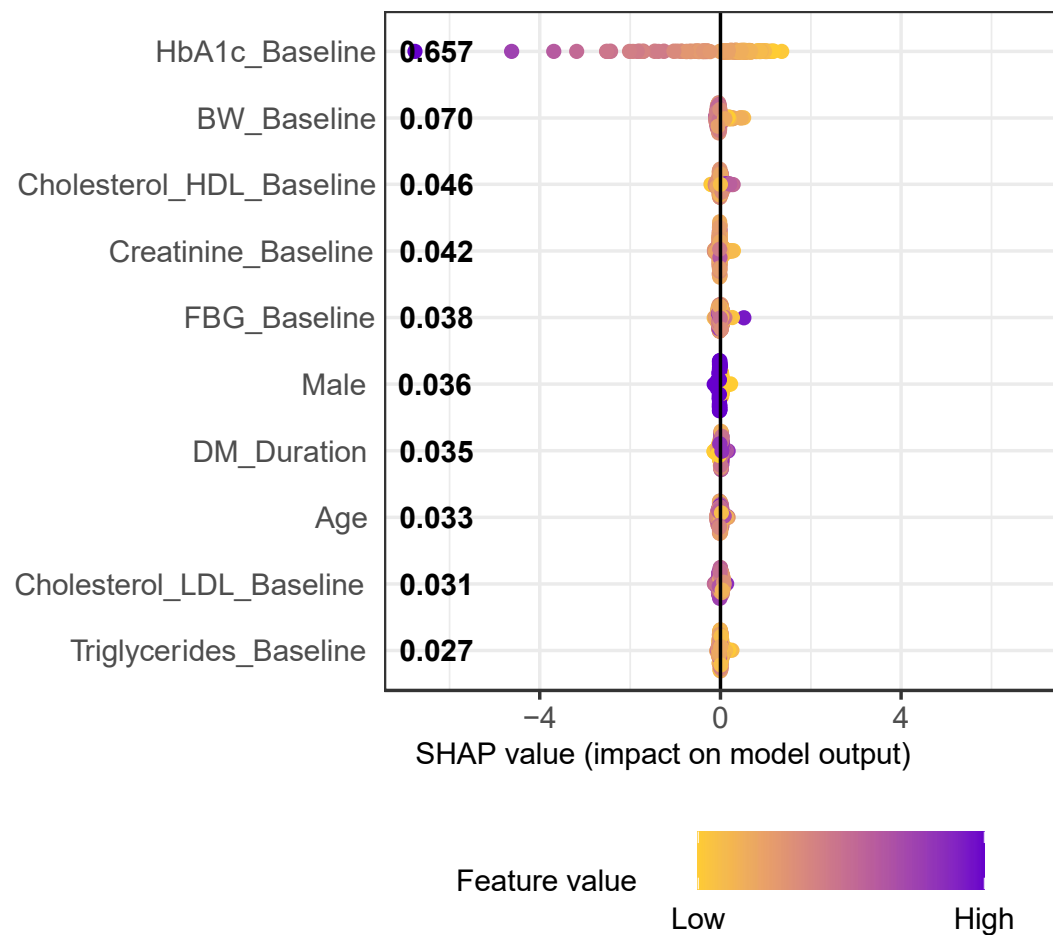

B)

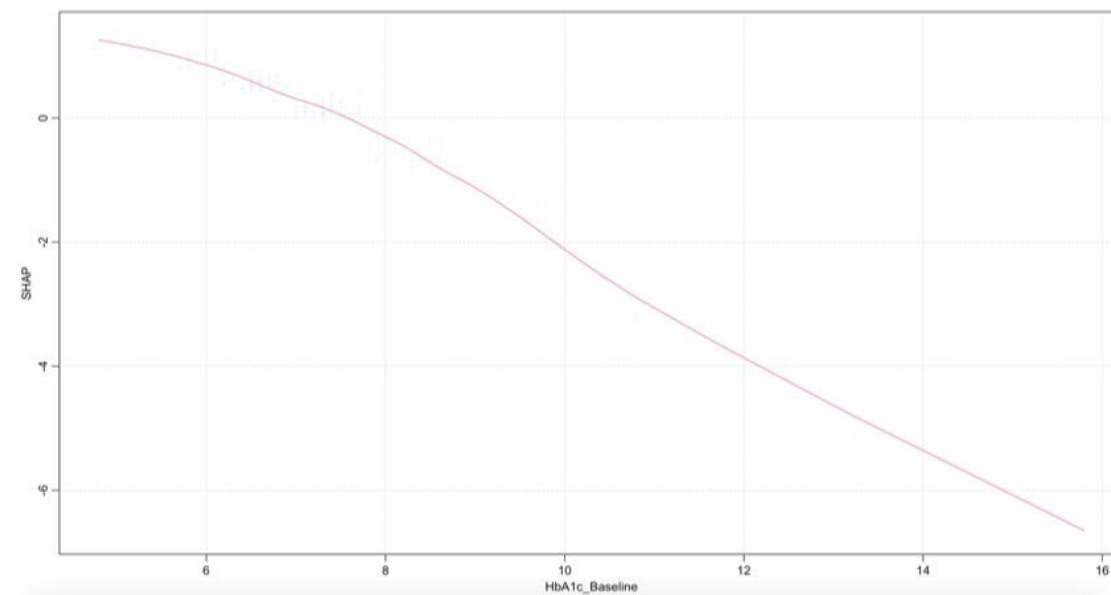

Supplement: Supplementary file 1 — Table S1. GLP‐1RA prescribed to included patients. Table S2. Stepwise multivariate regression model for prediction of HbA1c change from baseline. Table S3. Causal mediation analysis to assess the role of baseline HbA1c as a mediator of the effect of T2D phenotypes on HbA1c reduction. Table S4. Stepwise multivariate regression model for prediction of FBG change from baseline. Table S5. Stepwise multivariate regression model for prediction of BW change from baseline. Figure S1. Patients’ disposition. Figure S2. SHAP summary plot for the 10 most relevant features for change in HbA1c prediction (A) and dependence graph for HbA1c (B). Figure S3. SHAP summary plot for change in fasting blood glucose (FBG). Figure S4. SHAP summary plot for change in body weight (BW). [file DOM-27-6210-s001.zip › Supplementary Figure 2.pdf]
